# Transcriptome responses to temperature, water availability and photoperiod are conserved among mature trees of two divergent Douglas-fir provenances from a coastal and an interior habitat

**DOI:** 10.1186/s12864-016-3022-6

**Published:** 2016-08-26

**Authors:** Moritz Hess, Henning Wildhagen, Laura Verena Junker, Ingo Ensminger

**Affiliations:** 1 Forest Research Institute of Baden-Württemberg (FVA), Wonnhaldestrasse 4, D-79100 Freiburg i. Brsg., Germany; 2 Institute for Biology III, Faculty of Biology, Albert Ludwigs University Freiburg, Schänzlestrasse 1, D-79104 Freiburg i. Brsg., Germany; 3 Department of Biology, Graduate Programs in Cell & Systems Biology and Ecology & Evolutionary Biology, University of Toronto, 3359 Mississauga Road, Mississauga, ON L5L 1C6 Canada; 4 Present Address: Department of Forest Botany and Tree Physiology, Büsgen-Institute, Georg-August-University Göttingen, Büsgenweg 2, D-37077 Göttingen, Germany; 5 Present Address: Institute of Medical Biometry, Epidemiology and Informatics (IMBEI), University Medical Center Mainz, Obere Zahlbacher Strasse 69, 55131 Mainz, Germany

**Keywords:** Phenotypic plasticity, Transcriptome, Local adaptation, *Pseudotsuga menziesii*, Gene expression, Drought, Photoperiod, Heat, Natural environment, RNA-Seq

## Abstract

**Background:**

Local adaptation and phenotypic plasticity are important components of plant responses to variations in environmental conditions. While local adaptation has been widely studied in trees, little is known about plasticity of gene expression in adult trees in response to ever changing environmental conditions in natural habitats. Here we investigate plasticity of gene expression in needle tissue between two Douglas-fir provenances represented by 25 adult trees using deep RNA sequencing (RNA-Seq).

**Results:**

Using linear mixed models we investigated the effect of temperature, soil water availability and photoperiod on the abundance of 59189 detected transcripts. Expression of more than 80 % of all identified transcripts revealed a response to variations in environmental conditions in the field. GO term overrepresentation analysis revealed gene expression responses to temperature, soil water availability and photoperiod that are highly conserved among many plant taxa. However, expression differences between the two Douglas-fir provenances were rather small compared to the expression differences observed between individual trees. Although the effect of environment on global transcript expression was high, the observed genotype by environment (GxE) interaction of gene expression was surprisingly low, since only 21 of all detected transcripts showed a GxE interaction.

**Conclusions:**

The majority of the transcriptome responses in plant leaf tissue is driven by variations in environmental conditions. The small variation between individuals and populations suggests strong conservation of this response within Douglas-fir. Therefore we conclude that plastic transcriptome responses to variations in environmental conditions are only weakly affected by local adaptation in Douglas-fir.

**Electronic supplementary material:**

The online version of this article (doi:10.1186/s12864-016-3022-6) contains supplementary material, which is available to authorized users.

## Background

Plants continuously experience variations in environmental conditions on short (e.g. minutes to days) and long time scales (e.g. weeks or growing season). Short term responses to dynamic environments require strict physiological regulation and are known as phenotypic plasticity. On evolutionary timescales, responses to a specific environment may result in adaptation to local environment through genetic differentiation of populations, which is known as local adaptation [1]. As a consequence of local adaptation, plant populations frequently show the best growth performance or fitness in [2] or next to their habitat of origin [3] and outperform populations from distant habitats [4]. Potential genomic targets of adaptation to climate have been identified by linking single nucleotide polymorphisms (SNP) to local climate (e.g. in *Arabidopsis thaliana* [5], *Pinus taeda* [6], *Picea mariana* [7]) or by identification of associations among SNPs and traits that are known to co-vary with climatic clines, e.g. bud set and cold resistance (*Picea sitchensis* [8]), carbon isotope discrimination (*Pinus taeda* [9]) or cold hardiness (*Pseudotsuga menziesii* [10]).

With respect to the anticipated rapidly changing climate [11], forest trees, which have long generation times, need to adjust their metabolism in response to changing abiotic factors [12]. Global transcriptome analysis has been extensively used in studies with highly controlled conditions to characterize plasticity and diversity of gene expression metabolism in response to abiotic factors among different populations (e.g. in *Arabidopsis thaliana* [13–15], *Populus* [16], *Helianthus annuu*s [17], *Pinu*s spp. [18] or *Pice*a spp. [18, 19]. Plants do evolve in complex natural environments, and controlled conditions in a laboratory or greenhouse facility rarely represent the ever-changing complex conditions experienced by plants in natural environments [20]. Few studies investigated genome-wide gene expression responses to abiotic stimuli in natural environments [21, 19, 22–25] and studies in perennial, woody plants are exceptionally rare but see e.g. [19] on *Picea sitchensi*s, [22] on *Vitis vinifera* and [25] on *Populus euphratica*.

Douglas-fir is a commercially important *Pinaceae* tree species originating in western North America. Two subspecies, *Pseudotsuga menziesii* var. *menziesii* (Coastal Douglas-fir) and *Pseudotsuga menziesii* var. *glauca* (Interior Douglas-fir) diverged about 1 million years ago [26, 27] and cover a wide natural range with contrasting environmental conditions along the pacific coast and the Rocky Mountains, respectively. Local adaptation of Douglas-fir populations growing in defined geographical areas (provenances) has been shown in several studies [10, 28]. These characteristics make Douglas-fir an ideal model organism to study the effect of local adaptation on transcriptome responses to environmental conditions.

In this study we aimed to 1) identify transcriptome dynamics in field-grown adult Douglas-fir trees in response to temperature, water availability and photoperiod, 2) use overrepresentation analysis to reveal common functional themes in gene sets that respond to environmental factors, and 3) evaluate differences in transcriptome dynamics between provenances.

For this purpose we compared 50-year-old trees of two divergent Douglas-fir provenances originating from contrasting environments in British Columbia, Canada at two contrasting common garden field-sites in Southern Germany. To our knowledge, this is the first genome wide assessment of the effect of abiotic environmental factors on the transcriptome responses of heterogeneous, locally adapted populations of mature trees, grown in contrasting natural environments.

## Results

### Sequencing, alignment, quantification and functional annotation

**Fig. 1:**
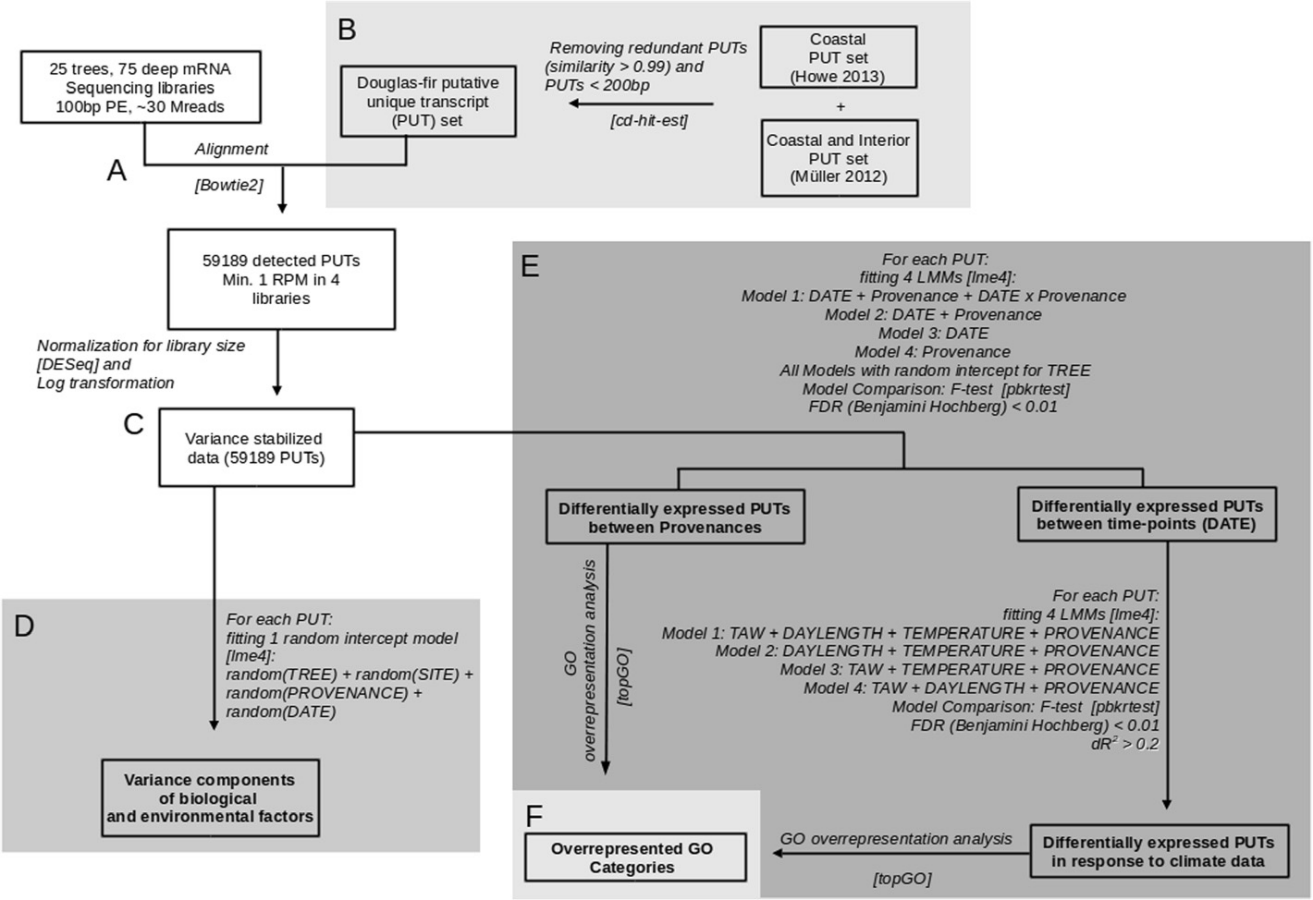
Analysis Pipeline. The sequence libraries were aligned (**a**) to a non-redundant set of two Douglas-fir PUT sets (**b**). After log-transforming the count data (**c**), linear random effect models were used to (**d**) detect sources of variation within the data. Differential expression between genotype and environment was investigated by linear mixed models (LMM) (**e**). Nested models were compared by an F-test with Kenward-Roger approximation [102]. Additionally the difference in AIC (dAIC) and R^2^ (dR^2^ ) was calculated. PUTs are only considered as responding to an environmental factor if the corresponding regressor increases the R^2^ by more than 0.2. Overrepresentation of GO categories within the differentially expressed PUTs was investigated (**f**)

We quantified transcript expression in 25 50-year-old Douglas-fir trees, growing at two common gardens (Wiesloch and Schluchsee) in southwestern Germany, during the growing season of 2010. 12 of these trees were from provenance Cameron Lake (LA), 13 were from provenance Salmon Arm (AR). A total of 75 RNA extracts from needle samples collected at noon in May, June, July and September at both field sites were sequenced on the Illumina HiSeq2000 (Fig. 1a). Reads were aligned to the set of 176753 non-redundant Douglas-fir putative unique transcripts (PUT) (Fig. 1b). The alignment yielded on average 33 million aligned reads (Mreads) per sequencing library. After excluding low abundant PUTs we detected 59189 PUTs (~34 %) of the 176753 PUTs present in the non-redundant set. Around 40 k PUTs were functionally annotated by alignment to the NCBI plant RefSeq data base (Table 1). GO annotations were identified for 34 k PUTs using BLAST2GO. 6330 PLAZA gene families were identified in the set of all detected PUTs.

**Table 1:** Annotation statistics of detected PUTs

Cluster type	Number of PUTs	Unique Annotations
PUTs detected with 1 RPM in at least 4 Libraries	59189	
hit in RefSeq	38248	21218
hit in *Picea glauca* ORFs	45867	14544
hit in *Pinus taeda* ORFs	42602	13842
hit in *Vitis vinifera*	38841	11263
hit in *Oryza sativa*	37408	11220
hit in *Arabidopsis thaliana*	34356	10762
B2GO annotation	34375	9062
PLAZA gene family	41175	6330

*RefSeq*: NCBI plant RefSeq peptide data base, *Picea glauca ORFs:* *Picea glauca* full length ESTs, *Pinus taeda ORFs:* de novo assembled ESTs which have been used to annotate the Pinus taeda genome, *Vitis vinifera:* *Vitis vinifera* peptide data base (PLAZA), *Oryza sativa:* *Oryza sativa* peptide data base (PLAZA), *Arabidopsis thaliana:* TAIR10 peptide data base, *B2GO:* GO annotations inferred by the BLAST2GO pipeline

### Variance components contributing to PUT expression variation

**Fig. 2:**
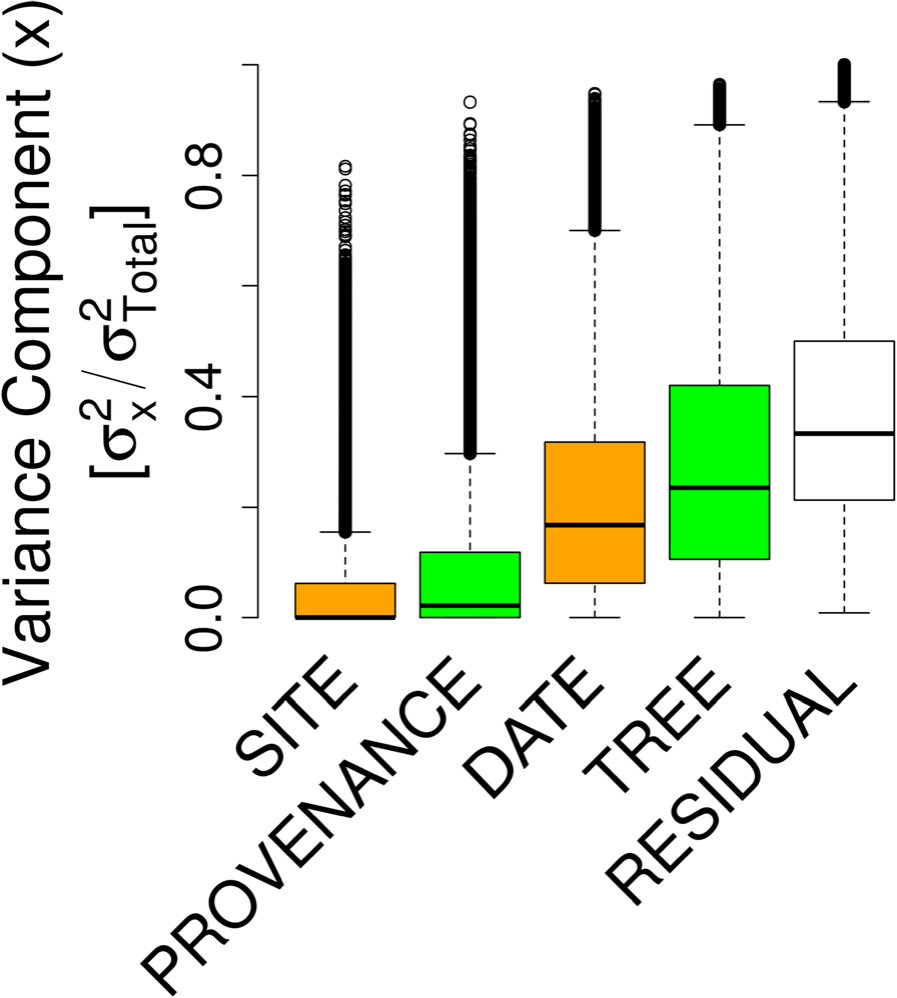
Variance components of biological and environmental factors. Proportion of total expression variation attributable to provenances (*PROVENANCE*), field sites (*SITE*), individual trees (*TREE*) sampling time point (*DATE*) for all detected 59189 PUTs. A linear random effects model that comprises all above mentioned random effects was fit to all PUTs using the R package lme4. Variance components for each random effect were extracted and divided by the sum of all variance components and the residual variance

In a first step we used a linear variance components model to estimate the contribution of the environment and the genotype to the variation in PUT expression. Expression variation driven by environment was investigated between the eight sampling time points across the two field sites (*DATE*) and between two field-sites (*SITE*). Expression variation driven by genotype was investigated between provenances (*PROVENANCE*) and between individual trees (*TREE*). The log-transformed number of reads that aligned to each PUT was used as the dependent variable in 59189 linear random effects models (Fig. 1d). For 25 % of all PUTs, at least 40 % of the variation in PUT expression was explained by *TREE* followed by *DATE* (30 %) and *PROVENANCE* (≥12 %) (Fig. 2). The least contribution to the variation in PUT expression was attributed to the field site (*SITE*; ≥7 %).

### Detection of differentially expressed PUTs between provenances and in response to environmental variation

**Fig. 3:**
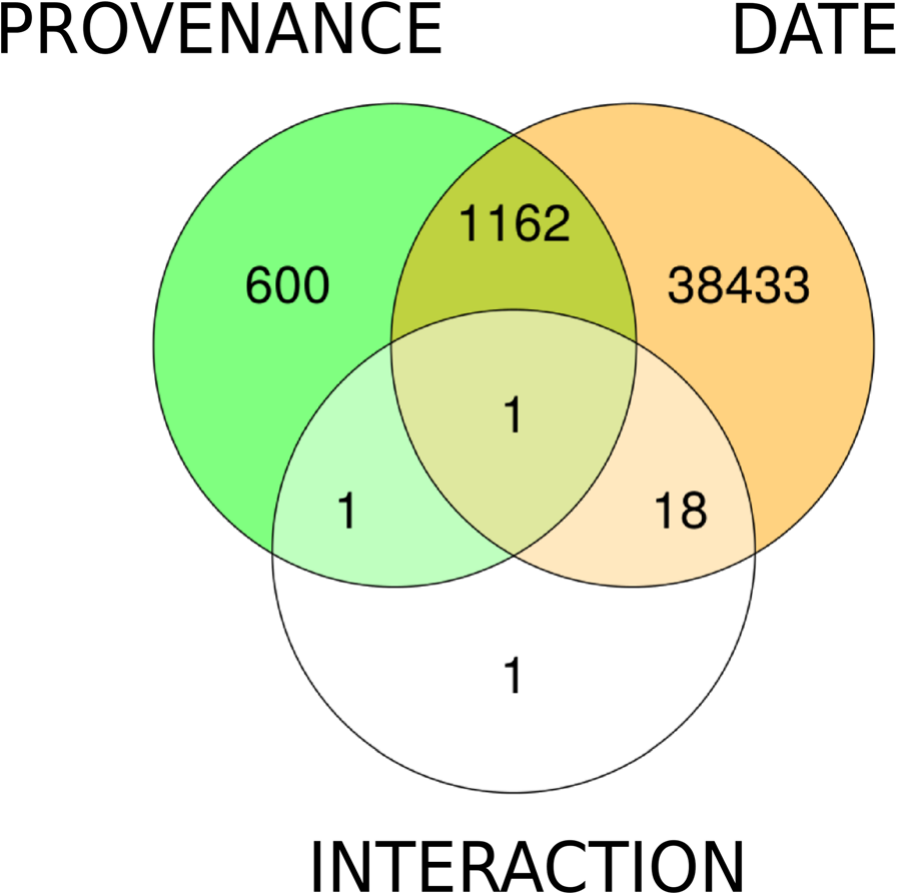
Differentially expressed PUTs between provenances and sampling time-points. Number of PUTs that are differentially expressed (FDR <0.01, F-test with Kenward-Roger approximation) between sampling time-points (*DATE*) or provenances and number of PUTs with significant interaction of provenances and sampling time-points

The effect of environment (E), genotype (G) represented by *PROVENANCE,* or their interaction (GxE) on PUT expression was further investigated using linear mixed models (see Fig. 1e). This analysis identified 1764 PUTs that were differentially expressed between the two provenances (*PROVENANCE*), 39614 differentially expressed PUTs between sampling time-points (*DATE*), and 21 PUTs that were affected by the interaction of provenance and environment (FDR <0.01, F-test with Kenward-Roger approximation) (Fig. 3).

**Fig. 4:**
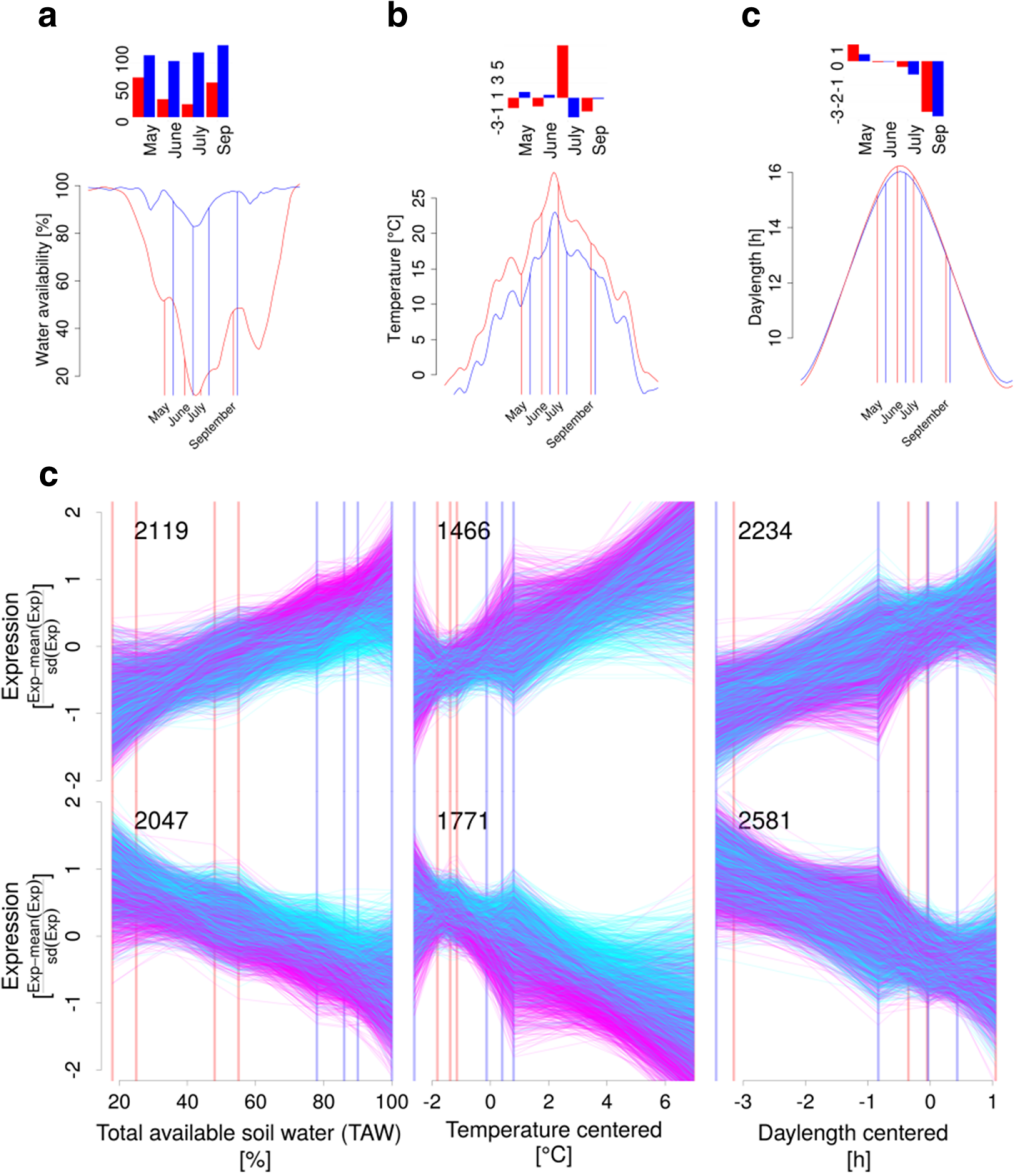
Effects of environmental factors on PUT expression. X-Y plots show soil water availability (**a**) temperature (**b**) and day length (**c**) during the course of the year. The corresponding bar plots show the values of the respective regressors *TAW*, *TEMPERATURE* and *DAYLENGTH* that were used in the linear models. Red and blue lines indicate the sampling time point in the common gardens in Wiesloch and Schluchsee, respectively. Curves were smoothed using loess transformation. Temperature shown in the bar plot (**b**) was centered to a 4 week running average while day length (**c**) was centered to the maximum day length of the year. Day length in May was transformed to positive values to mimic the directed developmental processes occurring during a growing period. **d** Standardized expression of PUTs that are significantly responding to *TAW*, *TEMPERATURE* or *DAYLENGTH* in Salmon Arm (*magenta*) and Cameron Lake (*cyan*) relative to the environmental parameters

Assuming that *DATE* integrates the effects of temperature, day length and water availability on PUT expression, we further analysed the direct effects of these environmental factors on PUT expression using a second set of linear mixed models (Fig. 1e). These models comprised proxies for temperature, day length and water availability. Since absolute temperature data were correlated with day length, temperature was detrended by subtracting the four weak running average from air temperature on the day of sampling resulting in the new variable *TEMPERATURE*. Day length was represented as deviation of photo period from the length of the day at solstice (*DAYLENGTH*) and total available soil water (*TAW*) was modeled using the forest water model WBS3 (Fig. 4a-c). We inspected correlation of all variables (Additional file 1: Figure S1) and observed no strong correlation between *TEMPERATURE*, *DAYLENGTH* and *TAW*. However *SITE* was strongly correlated with *TAW* (Additional file 1: Figure S1) and since the variance components analysis revealed that not much expression variance can be attributed to differences between the field sites (Fig. 2) *SITE* was omitted from this second set of models.

*TAW* (Fig. 4a) was generally lower at the field site Wiesloch during all sampling time-points (*DATE*) and low *TAW* was often accompanied by higher temperature (Fig. 4b). The highest temperatures at both sites were recorded in July, when temperature exceeded the four week average by 7 °C in Wiesloch. *DAYLENGTH* in September did contrast with May, June and July, with day length in September being 3–4 h shorter than during May, June and July (Fig. 4c).

Further analysis of the PUTs that responded to *TAW*, *TEMPERATURE* or *DAYLENGTH* revealed the expression profile in response to each of the environmental factors (Fig. 4d). The relationship of expression levels of PUTs to any of the three environmental factors indicated a highly coordinated response over the entire range of *TAW*, *TEMPERATURE* or *DAYLENGTH* (Fig. 4d). The expression profile of PUTs with a positive response clearly contrasted with the expression profile of PUTs with a negative response to each of the environmental factors. Increases in *TAW* corresponded with an increased expression of 2119 PUTs (FDR <0.01, F-test with Kenward-Roger approximation, dR^2^ >0.2) and a decreased expression of 2047 PUTs. When *TEMPERATURE* increased, we observed increased expression of 1466 PUTs versus 1771 PUTs showing the opposite response. An increase in *DAYLENGTH* corresponded with increased expression of 2234 PUTs compared to 2581 PUTs with decreased expression.

**Fig. 5:**
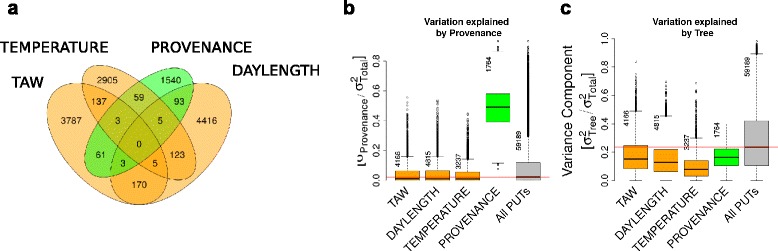
Variation in expression response to environment among provenances. **a** Number of PUTs that are differentially expressed between provenances or responding to *TEMPERATURE*, *DAYLENGTH* or *TAW*. **b** Expression variation among provenances of PUTs that respond to environmental factors or are differentially expressed between provenances compared to all detected PUTs. **c** Expression variation among trees of PUTs that respond to environmental factors or are differentially expressed between provenances compared to all detected PUTs. The red line in **b** and **c** indicates the median of expression variation in all detected PUTs. Numbers at the whiskers of each box plot indicate the number of PUTs within a category

The specific response to environmental factors (Fig. 4d) was also reflected by only 446 PUTs out of the more than 12 k differentially expressed PUTs that were effected by more than one environmental factor (Fig. 5a).

In addition to the low number of PUTs with provenance by environment interaction, only 224 PUTs out of the 1764 PUTs that were differentially expressed between provenances were also differentially expressed in response to at least one of the three environmental factors included in our analysis (Fig. 5a). This set of 224 PUTs included 101 PUTs differentially expressed between provenances and in response to *DAYLENGTH*, 67 PUTs differentially expressed between provenances and in response to *TAW*, and 62 PUTs differentially expressed between provenance and in response to *TEMPERATURE*.

The low number of PUTs that responded to climate and were differentially expressed between provenances coincided with reduced expression variation of these PUTs among provenances (Fig. 5b) and trees (Fig. 5c). This effect is particularly pronounced for PUTs that responded to *TEMPERATURE*.

### Gene Ontology categories overrepresented in differentially expressed PUTs

An overrepresentation analysis was performed to identify Gene Ontology (GO) categories in the differentially expressed PUTs that showed a response to environmental conditions or showed differences between the provenances. Using Fisher’s exact test (*P*-value < 0.01, minimum 10 differentially expressed PUTs within a GO category) we identified 63 GO categories that were overrepresented in higher expressed PUTs when *TAW* was low, in contrast to 83 GO categories that were overrepresented when *TAW* was high. For *TEMPERATURE* we identified 54 GO categories that were overrepresented in higher expressed PUTs when *TEMPERATURE* was high and 143 GO categories that were overrepresented when *TEMPERATURE* was low. For *DAYLENGTH* we identified 101 GO categories that were overrepresented in higher expressed PUTs when *DAYLENGTH* was longer, compared to 80 GO categories that were overrepresented when *DAYLENGTH* was shorter. A comparison of GO ontologies between the two provenances revealed 117 GO categories that were overrepresented in PUTs that were stronger expressed in provenance Cameron Lake compared to one single GO category that was overrepresented in PUTs that were stronger expressed in provenance Salmon Arm (Additional file 2: Table S1).

**Fig. 6:**
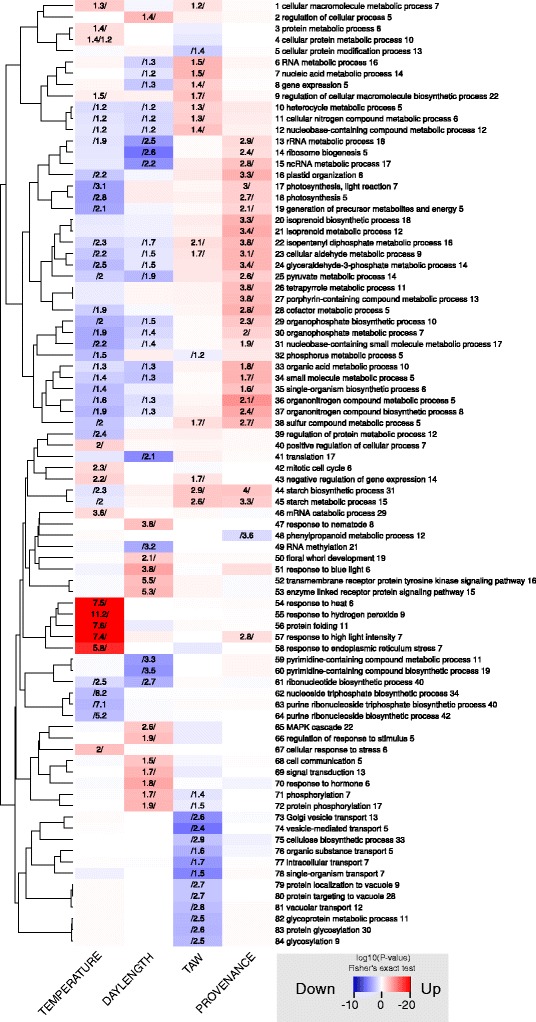
GO term overrepresentation analysis of PUTs that respond to environment or are differentially expressed between provenances. The top 20 GO categories that were significantly overrepresented within PUTs that respond to *TEMPERATURE*, *TAW*, *DAYLENGTH* or *PROVENANCE* are shown. Redundant GO categories have been removed using REVIGO [103] with default parameters. Numbers in columns indicate the amount of overrepresentation which is the number of significant PUTs within a GO category divided by the number of expected PUTs within a GO category and are only shown for significant overrepresentation of a GO category (Fisher’s Exact Test, *P* < 0.01). The *p*-value of Fisher’s exact test is indicated by color intensity. Red color indicates high overrepresentation of a GO category within PUTs that are positively correlated with *TEMPERATURE* and *DAYLENGTH,* negatively correlated with *TAW* or stronger expressed in Cameron Lake (*PROVENANCE*). Blue color indicates high overrepresentation of GO categories within PUTs that are negatively correlated with *TEMPERATURE* and *DAYLENGTH,* positively correlated with *TAW* or stronger expressed in Salmon Arm (*PROVENANCE*). The dendrogram indicates the similarity of GO categories in terms of shared PUTs among GO categories, expressed by Cohen’s Kappa. Numbers behind GO categories indicate the hierarchy of the GO graph, while more basal terms have smaller numbers

For each regressor and direction of expression we chose the 20 most overrepresented GO categories and investigated the biological functions of PUTs within each GO category (Fig. 6). We investigated the best hit in the *Arabidopsis thaliana* peptide data base (TAIR10) and in the NCBI RefSeq data base of PUTs that we observed within the overrepresented GO categories. The best hits in the TAIR10 data base as well as in the NCBI RefSeq data base are listed in Additional file 3: Table S2. Hits were ranked according to their absolute log10 transformed *P*-value from the test of differential expression. All functional descriptions in the next sections that are not followed by a citation were retrieved from the TAIR data base.

#### Biological functions of differentially expressed PUTs that respond to TAW

GO categories related to starch metabolism, e.g. “starch biosynthetic process”, “starch metabolic process”, and nucleic acid metabolism, e.g. “RNA metabolic process”, “nucleic acid metabolic process”, “gene expression”, “regulation of cellular macromolecule compound metabolic process” and “nucleobase-containing compound metabolic process” (Fig. 6; rows 44, 45, 6, 7, 8, 9, 12; Additional file 2: Table S1 - *TAW_*Down) were highly overrepresented in PUTs that were higher expressed when *TAW* was low (*P*-value Fisher’s exact test < 1e-04). PUTs within the GO categories related to nucleic acid metabolism included homologs of the protein kinase WEE1 (AT1G02970; Additional file 3: Table S2 - *TAW*_Down; rank 1) which negatively regulates the entry into mitosis [29], the CDC2 related kinase AFC1 (AT3G53570; rank 6), the WUSCHEL-related homeobox gene family member WOX13 (AT4G35550; rank 13), the apurinic endonuclease-redox protein ARP (AT2G41460; rank 15) which is involved in DNA repair, the splicing factor RSZ22 (AT2G24590; rank 24), the NAC domain transcription factor ANAC002 (AT1G01720; rank 28) whose mRNA levels increase in response to abscisic acid in *Arabidopsis thaliana* or the zinc-finger proteins CCCH20 and CCCH49 (AT2G19810; ranks 33, 187) that are involved in RNA binding in the response to osmotic stress [30].

Within the GO categories related to starch metabolism we identified homologs of the phosphoglucomutase PGMP (AT5G51820; rank 2), which controls photosynthetic carbon flow, the beta amylase BAM3 (AT4G17090; ranks 20, 34), the starch branching enzyme SBE2.2 (AT5G03650; rank 21), or the fructose 1–6 bisphosphatase FBP (AT1G43670; rank 41).

GO categories related to intracellular transport, e.g. “Golgi vesicle transport”, “vesicle mediated transport or “intracellular transport” (Fig. 6; rows 73, 74, 77; Additional file 2: Table S1 - *TAW* Up), were highly overrepresented in PUTs that were higher expressed when *TAW* was high (*P*-value Fisher’s exact test < 1e-08). Within these categories we detected homologs of the vacuolar sorting receptor VSR6 (AT1G30900; Additional file 3: Table S2 - *TAW* Down; ranks 4, 8, 487), the MATE efflux protein (AT1G51340; rank 5), the membrane trafficking proteins SYTA and SYTB (AT2G20990 and AT2G20080; rank 6 and rank 14), the UDP glucose / UDP galactose transporter UTr7 (AT4G31600; rank 11), or the sucrose transporter SUC3 (AT2G02860; ranks 19, 181, 430, 590).

#### Biological functions of differentially expressed PUTs that respond to TEMPERATURE

GO categories related to oxidative and heat stress, e.g. “response to heat”, “response to hydrogen peroxide” (Fig. 6; rows 54, 55; Additional file 2: Table S1 - *TEMPERATURE* _Up) were highly overrepresented (*P*-value Fisher’s exact test < 1e-16) in PUTs that were stronger expressed when *TEMPERATURE* was high. PUTs within these categories were homologs to heat shock proteins (Additional file 3: Table S2 - Temperature Up; e.g. ranks 13, 16, 17, 22, 27, 31). Other proteins identified are involved in the acclimation to heat, e.g FKBP62 which is engaged in thermotolerance (AT3G25230; ranks 5,6) [31] or the multiprotein bridging factor MBF1C (AT3G24500; rank 138) which interacts as a co-factor with bZip transcription factors [32].

GO categories related to control of gene expression, e.g. “regulation of cellular macromolecule biosynthetic process”, “negative regulation of gene expression” (Fig. 6; rows 9, 43) were also overrepresented. These categories comprised homologs of ethylene responsive transcription factors with ERF/AP2 domain (AT1G19210; ranks 116, 204, 281 - AT5G21960; ranks 119, 161, 178, 181 - AT1G74930; rank 271 - AT5G11590; rank 258) which are involved in various stress responses in plants [33]. We also identified homologs of HSF2A (AT2G26150; rank 24) which is an important heat shock transcription factor [34]. In addition to genes directly involved in control of gene expression, we also observed homologs of genes which are involved in chromatin modification [35], such as the histone methyltransferase SUVH4 (AT5G13960; rank 14) and OTLD1 (AT2G27350; rank 155) [36].

GO categories “photosynthesis, light reaction”, “photosynthesis” and “generation of precursor metabolites and energy” (Fig. 6; rows 17, 18, 19) were highly overrepresented (*P*-value Fisher’s exact test < 1e-06) in PUTs showing a negative response to high *TEMPERATURE*. Within these categories, we observed homologs of the triose phosphate/3-phosphoglycerate translocator APE2 (AT5G46110; Additional file 3: Table S2 - “*TEMPERATURE_* Down”; rank 2), the large subunit of ADP-glucose pyrophosphorylase ADG2 (AT5G19220; rank 28) which catalyzes the first rate limiting step in starch biosynthesis, the cytosolic malate dehydrogenase c-NAD-MDH1 (AT1G04410; ranks 22,100) or the chloroplast protein phosphatase TAP38/PPH1 (AT4G27800; ranks 62, 241) which dephosphorylates the light harvesting complex of photosystem II [37].

#### Biological functions of differentially expressed PUTs in response to DAYLENGTH

GO categories related to signalling and regulation, e.g. “regulation of cellular process”, “response to blue light”, “regulation of response to stimulus”, “signal transduction”, “response to hormone” (Fig. 6; rows 2, 51, 66, 69, 70) were highly overrepresented (*P*-value Fisher’s exact test < 1e-05) in PUTs that were higher expressed under long day conditions. PUTs within these categories were homologous to the floral homeotic protein APETALA2 (AT4G36920; Additional file 3: Table S2 - “*DAYLENGTH* Up”; ranks 3,4), the homeobox leucine zipper proteins HB1 (AT3G01470; rank 9), HB5 (AT5G65310; rank 1) and HB6 (AT2G22430; rank 2), the flavonoid 3 hydroxylase 2 CYP75B1 (AT5G07990; ranks 11,177, 247), the lipoxygenase LOX5 (AT3G22400; ranks 14, 116), the myb family transcription factor MYB33 (AT5G06100; rank 26, 43) or the inositol triphosphate 5 phosphatase 2 5PTASE2 (AT4G18010; ranks 12, 29, 33) which regulates growth in seedlings [38].

Within the above mentioned GO categories we also observed homologs of members of the flowering control network which is tightly coupled to sensing changes in the duration of photoperiod [39]. Examples are GIGANTEA (GI; AT1G22770; ranks 311, 317, 417), flowering locus t (FT; AT1G65480; ranks 185, 309), phytochrome B (AT2G18790; rank 327) or leafy (LFY; AT5G61850; rank 103).

GO categories “ribosome biogenesis” and “translation” (Fig. 6; rows 14, 41) were highly overrepresented (*P*-value Fisher’s exact test < 1e-09) in PUTs that were higher expressed when daylength decreased. However the PUTs that were most strongly differentially expressed in these conditions were found within the weakly overrepresented GO categories related to gene expression, e.g. “nucleic acid metabolic process” or “gene expression”, and pyruvate metabolism, e.g. “pyruvate metabolic process” (Fig. 6; rows 7, 8, 25). PUTs within these GO categories were homologous to the small hydrophobic protein RCI2A (AT3G05880; Additional file 3: Table S2 - “*DAYLENGTH_*Down”; rank 2), the histidine kinase phytochrome PHYE (AT4G18130; rank 4), the GATA transcription factor GATA12 (AT5G25830; rank 5), a member of the DREB subfamily A2 (AT5G05410; rank 9), the enolase ENO1 (AT1G74030; ranks 13, 14), the thylakoid protein PSB29 (PSB29, AT2G20890; rank 24) [40] or the phosphatidylglycerol phosphate synthase PGP1 (AT2G39290; rank 42) which is involved in cold acclimation [41]. In addition we observed many NAC domain containing transcription factors (e.g. AT4G29230; rank 27 – AT1G01720; rank 34 – AT4G29230; rank 51).

PUTs with homology to RCI2A were also found in GO category “osmotic stress” which was overrepresented as well (Additional file 2: Table S1 - *DAYLENGTH* Down). Within this category we identified homologs of the cold regulated proteins COR314 and COR413 (AT1G29390; rank 6 – AT1G29395; rank 10), the alcohol dehydrogenases ADH and ADH2 (AT1G77120; ranks 11, 15 – AT5G43940; rank 16), RCI2B (AT3G05890; rank 37) or the NADPH dependent aldo-keto reductase AKR4C9 (AT2G37770; rank 40).

#### Biological functions of PUTs differentially expressed between provenances

GO categories related to pigment biosynthesis or photosynthesis, e.g. “cofactor metabolic process”, “isoprenoid metabolic process”, “photosynthesis” or “plastid organization” (Fig. 6; rows 28, 21,18, 16) were highly overrepresented (*P*-value Fisher’s exact test < 1e-06) in PUTs that were higher expressed in Cameron Lake than in Salmon Arms.

Upregulated PUTs observed within the GO categories related to photosynthesis included homologs of the blue light receptor NPL1 (AT5G58140; Additional file 3: Table S2 - “*PROVENANCE_*Up”; rank 8) which mediates stomatal opening and chloroplast movement [42], the protease DEG1 (AT3G27925; rank 9) which is targeted to the chloroplast to repair damages of the photo system [43], the chlorophyll A oxigenase CAO (AT1G44446; rank 40) which enhances photosystem efficiency by increasing the antenna size of photosystems [44], or the small subunit of RUBISCO (AT1G67090; rank 223). PUTs within GO categories related to pigment biosynthesis were homologous to the phytoene synthase PSY (AT5G17230; rank 12), the NADPH thioredoxin reductase NTRC (AT2G41680; rank 16), the ferrochelatase 2 FC-II (AT2G30390; ranks 35,155), or the zeaxanthin epoxidase ABA1 (AT5G67030; rank 79) which converts the photoprotective xanthophyll zeaxanthin into antheraxanthin and violaxanthin [45].

Only the GO category “phenylpropanoid metabolism” was overrepresented in PUTs that were higher expressed in Salmon Arm (Fig. 6; row 48). Within this GO category we identified PUTs that were homologous to the most basal enzymes of the phenylpropanoid pathway, e.g. the 4-coumarate ligase 4CL (AT1G20510; Additional file 3: Table S2 - “*PROVENANCE_*Down”; ranks 1–5), the cinnamate-4 hydroxylase C4H (AT2G30490; rank 17), the O-methyltransferase OMT1 (AT5G05170; rank 12) or the chalcone synthase TT4 (AT5G13930; rank 7).

## Discussion

### A large part of the Douglas-fir transcriptome responds to variations in environmental conditions in the field

We have investigated transcript expression in needles of adult Douglas-fir trees growing under natural field conditions. We assessed differences in transcriptome dynamics in response to variations in environmental conditions but also variations in transcript abundance among individual trees and among two differentially adapted provenances. The alignment of our RNA-Seq data to our non-redundant Douglas-fir PUT set using the unigene catalogues of [46, 47] allowed identification of 59189 expressed PUTs. These 60 k PUTs correspond to 14539 unique hits in the *Picea glauca* gene catalogue [48]. We assume that these unique hits correspond to an equivalent number of unique gene loci which is in concordance with the number of expressed genes in *Pinaceae* needle tissue [49]. Despite stringent cut-off values (FDR <0.01, dR^2^ >0.2), many *Picea* homologs were differentially expressed in response to variations in environmental conditions: 15 % for *TAW,* 10 % for *TEMPERATURE*, 16 % for *DAYLENGTH* and 82 % for *DATE*. A recent gene expression study conducted in *Pinaceae* detected 5794 of 14691 (FDR <0.01) ortholog sequences among *Picea* and *Pinus* to be differentially expressed in response to environmental conditions in a growth chamber [18], this being in the range of our estimates. The most comprehensive transcriptome analysis performed in *Oryza sativa* under field conditions estimated that 43 % of all expressed genes respond to temperature, radiation and other macroenvironmental factors [50]. Richards et al. [23] detected variation in expression over time in almost all genes in two *Arabidopsis* accessions that were grown in a natural environment. This indicates that a large part of expressed genes in leaf tissue responds to variations in natural environmental conditions.

### PUTs that are differentially expressed in response to TEMPERATURE and DAYLENGTH reveal homology to genes controlled by heat stress and photoperiod

High temperature at the field sites clearly shaped gene expression in Douglas-fir needles. We observed stronger expression of PUTs that are related to heat shock proteins and other heat stress related proteins like MBF1 when *TEMPERATURE* was high, e.g. the ascorbate peroxidase APX2 or HSFA2 [34]. In addition we observed stronger expression of PUTs related to ERF/AP2 family transcription factors that are also well known to be stronger expressed in response to stress [51]. In contrast, PUTs related to photosynthetic activity, sugar and energy metabolism were weaker expressed when *TEMPERATURE* was high. Notable examples are PUTs related to the triosephosphate/3-phosphoglycerate translocator APE2 which is a key component in transporting assimilated carbon from the chloroplast into the cytosol, or the ADP-glucose pyrophosphorylase ADG2 which is important in starch biosynthesis. Reduced expression of genes involved in photosynthesis in response to temperature stress has already been described by [52] in *Arabidopsis* and by [53] in *Arabidopsis*, *Populus* and *Glycine*. Taken together, the observed gene expression pattern suggests a highly conserved response to temperature in several herbaceous plants and trees such as poplar, and based on our data also in Douglas-fir.

Day length controlled more than 16 % of the transcriptome. Major GO categories that were overrepresented in PUTs that were more abundant when day length was long were “meristeme development” or “response to hormone stimulus” (Fig. 6; rows 8–11). Upon deeper analysis, we observed many members of the gene network that controls flowering in angiosperms [39].

Exposure to short day length resulted in a complex response of the transcriptome. PUTs that were higher expressed when *DAYLENGTH* was low displayed homology to proteins involved in cold acclimation like RCI2 -A and -B [54], ADH [55] or the NADPH dependent aldo-keto reductase AKR4C9 [56]. Preparation for cold acclimation is associated with osmotic stress [57]. Thus, it is not unexpected that we observed PUTs with homology to DREB2 [58] or NAC domain containing transcription factors that are known to be induced by dehydration stress [51].

The most comprehensive study of cold acclimation in a natural environment has been conducted in *Picea sitchensis* seedlings where the authors contrasted gene expression during October, November and December with late summer gene expression during August [19]. Several transcripts with homology to proteins that are known to be involved in the adaptation to cold were identified in this study. Although their sampling time-points did only partially overlap with ours, over 40 % of the best hits in the *Arabidopsis thaliana* peptide data base observed in [19] were also observed in the differentially expressed PUTs in response to *DAYLENGTH*. This consistent pattern is remarkable as we used a different measurement method, used strict filter criteria to classify PUTs as significant, and used adult individuals of a different conifer species. Examples for overlapping annotations are GI or RCI2-A. Interestingly, neither [19] nor our study detected stronger expression of CBF/DREB transcription factors which are known to be essential for acclimation to low temperatures in *Arabidopsis thaliana* [59, 60] when day length decreases.

### Reduced *TAW* induced expression of transcripts related to starch metabolism, a conserved response to reduced water availability in plants

In response to low *TAW*, GO categories related to nucleic acid and starch metabolism were overrepresented, while GO categories related to intracellular transport were overrepresented when *TAW* was high (Fig. 6; Rows 6–12, 44, 45 and 73–81 respectively). Although no GO categories related to osmotic stress were significantly overrepresented we observed upregulation of individual PUTs that are involved in osmoregulation. We observed homologs of the osmosensor HK1 (AT2G17820; rank 179) which is higher abundant in *Arabidopsis* when osmolarity is especially high or low [61], or to aquaporins which are involved in handling osmotic stress [62]. In particular the aquaporins TIP1 (AT2G36830; rank 140), PIP2.8 (AT2G16850; rank 234), and PIP2.2 (AT2G37170; rank 279) were identified. Compared to the response to reduced day length, fewer specific indicators for osmotic stress were identified. However, we identified several homologs representing general stress responses when TAW was low. We observed PUTs with homology to the AP2/ERF domain containing transcription factors ERF-1 (AT4G17500; Additional file 3: Table S2 - *TAW*_Down; rank 70), DEAR2 (AT5G67190; rank 146) and EBP (AT3G16770; rank 224). AP2/ERF domain containing transcription factors are involved in general stress responses but are also involved in osmotic stress [51]. In addition we observed homologs of the copper/zinc superoxide dismutase CSD1 (AT1G08830; rank 40) which is known to be expressed in drought stressed plants [63], the NADPH dependent thioredoxin reductase NTRC (AT2G41680; rank 46) and the protochlorophyllide oxidoreductase PORA (T5G54190; rank 81) which protect the chloroplast against oxidative damage [64, 65]. These annotations are indicators for a certain amount of stress that the trees were confronted with during June and July at the site Wiesloch when *TAW* was low.

Transcription factors that are typically not known to be involved in the response to reduced water availability were also identified in the PUTs that were stronger abundant when water availability was low. Examples are the helix-loop-helix protein CIB (AT1G26260; rank 77), the transcriptional repressor MYB4 (AT4G38620; ranks 124, 132, 329, 334) which is involved in the response to UV-B [66] or SIG5 (AT5G24120; rank 171) which is expressed in response to high light [67].

In contrast to the responses to heat and changes in photoperiod, gene expression responses to decreases in water availability are less uniform among different experiments conducted in the same species and organ [68]. This is because the experimental manipulation of water stress is far more difficult to control than the manipulation of photoperiod and temperature. Nevertheless, an increase in starch metabolism under conditions of water shortage was also observed under conditions of mild drought stress in *Arabidopsis thaliana* [14]. A recent meta-analysis also revealed that enhanced expression in response to drought is conserved among species including *Oryza sativa*, *Arabidopsis thaliana*, *Triticum aestivum* or *Glycine max* [69]. Pinheiro and Chaves [68] and Prasch and Sonnewald [52] did also report downregulation of genes related to intracellular transport in *Arabidopsis thaliana* which is consistent with our data. We also observed overrepresentation of the GO category “cell growth” in PUTs that were higher expressed when *TAW* was high (Additional file 2: Table S1 - *TAW*_Up). Thus, our results indicate reduced cell growth and proliferation when water availability is low. This has also been reported for *Arabidopsis thaliana* by [70] and [14]. Indicators for the reduced growth are homologs of WEE1 which negatively regulates the entry into mitosis [29] that were higher expressed when *TAW* was low.

In contrast to the response to high *TEMPERATURE*, no GO categories related to photosynthesis were overrepresented in PUTs that were weaker expressed when *TAW* was low. This indicates no effect of low water availability on photosynthetic related gene expression. This phenomenon has also been reported by [14] in *Arabidopsis thaliana*.

### The two provenances Cameron Lake and Salmon Arm differ in constitutive expression of transcripts related to photosynthesis

The effect of environment on the global transcript expression was high, nonetheless expression differences between the provenances were rather small, since only 1764 PUTs were differentially expressed between the provenances. The weak expression differences between the provenances were not only indicated by the low number of differentially expressed PUTs, but also by the generally small variation in PUT expression levels among provenances (Fig. 5b) and a high variation in expression among trees (Fig. 5c). These findings are consistent with reports by [19] who detected only about 900 differentially expressed transcripts between provenances from contrasting habitats in *Picea sitchensis*, or by [71] who reported weak genetic variation in the metabolite abundance among multiple Douglas-fir families.

Despite their small number, the transcripts that are differentially expressed between the two provenances are likely responsible for differences in adaptive traits. For example, PUTs that were observed in higher abundance in Cameron Lake were related to photosynthesis. Examples are homologs of the serine/threonine kinase NPL1 (also known as PHOT2), which can act as a blue light photoreceptor and is involved in controlling of stomatal opening [72]. Overexpression of NPL1 resulted in enhanced photosynthetic activity and growth in Arabidopsis thaliana [42]. Other examples are the protease DEG1, which is targeted to the chloroplast to repair damages of the photosystems [43] or the chlorophyll A oxygenase CAO which enhances the efficiency of the photosystems by increasing the antenna size of photosystems [44]. The higher expression of these genes might translate into a generally higher photosynthetic activity in Cameron Lake.

Moreover, it has been frequently reported from common garden experiments that water use efficiency (WUE) is higher in coastal than in interior Dougals fir [73, 74] and [75]. This is a counterintuitive observation, since one would expect that interior Douglas fir provenances from higher altitudes would generally be better adapted to episodic water limitations, which is often associated with increased WUE. Aitken et al. [74] and Zhang et al. [75] suggested that the location of the experimental sites must have an influence on WUE. However, the observation that NPL1/PHOT2, which controls stomatal opening and hence WUE, is higher expressed in the coastal provenance Cameron Lake indicates that higher WUE in a coastal Douglas fir provenance might result from an increased ability for regulating stomatal behavior and mediating higher WUE.

### Interactions of provenance and environment

The effect of the interaction among provenance and *DATE* which represents the genotype by environment interaction (GxE), was surprisingly low with only 21 PUTs showing a response (Fig. 3, Additional file 4: Figure S2). Only three of these PUTs were homologous to *Arabidopsis thaliana* genes: a calmodulin binding protein (AT2G26190), an Armadillo repeat protein (AT4G34940) and a subunit of the cytochrome oxidase COX1 (ATMG01360). Due to the small extent of the GxE effect we did not further investigate interactions among provenances and the individual environmental regressors (*TAW*, *TEMPERATURE* and *DAYLENGTH*) because we expected those interactions to be even weaker. Weak GxE effects on the transcriptome dynamics were also observed in field-grown seedlings of *Arabidopsis thaliana* from contrasting habitats [23]. In addition, there is also evidence that transcript levels of only a small number of genes are influenced by eQTL x environment interactions in *Arabidopsis thaliana* [76].

PUTs that responded to environment (*TAW, DAYLENGHT, TEMPERATURE*) showed a lower variation of PUT abundance among provenances (Fig. 5b) and trees (Fig. 5c) compared to all detected PUTs. Thus, the small GxE effect in our data from field-grown adult Douglas-fir trees suggests that plastic transcriptome responses to variations in environmental conditions are strongly conserved both at the tree and provenance level. These findings are supported by results from [18] who observed that 74 % of the genes that respond to variations in environmental conditions in a growth chamber experiment also displayed conserved expression patterns in *Picea* and *Pinus* despite the large divergence time of both species (> 140 million years). Most notably, in our experiments the expression of PUTs that responded to *TEMPERATURE* varied less among provenances and trees compared to all other environmental factors (Fig. 5b, c). Previous experiments performed in growth chambers comparing *Glycine max, Arabidopsis thaliana* and seedlings of *Populus trichocarpa* revealed that transcriptome dynamics in response to high temperature are conserved across these angiosperm species [53]. Nevertheless, the observation that there is such a small variation in the response to high temperature among mature field grown trees and provenances of a conifer species is striking and highly relevant for foresters for adapting forests to climate change. Overall, the small GxE effects indicate that local adaptation has a rather small effect on the ability of Douglas-fir trees to modulate gene expression and their ability to deal with novel climates. It seems that differences in gene expression between the provenances (factor *PROVENANCE*), indicated by 1764 differentially expressed PUTs and overrepresentation of GO categories including photosynthesis and secondary metabolism, by far outweigh the importance of GxE effects in the two provenances included in our study. Nevertheless we only investigated a rather small number of genotypes, and only over one growing season. Thus, it would be important to assess if the genes that contributed here to the plastic response to environment between the two provenances will be also involved among larger groups of populations and larger temporal scales.

## Conclusions

Whole transcriptome responses to natural and highly variable environmental conditions were studied in adult Douglas-fir trees representing two populations from contrasting habitats. We investigated the correlation of transcript abundance with regressors that represented high temperature, photoperiod and water availability. Functional annotation and overrepresentation analysis revealed that Douglas-fir transcript regulation was similar to other species, indicating the high conservation of transcript expression in response to environmental cues. Thus our data set represents a rich repository of validated transcriptional responses to the main abiotic parameters of a natural and highly variable environment.

Almost no transcripts with divergent plastic expression responses between the provenances were observed. In addition, the transcripts that responded to environmental cues varied less among trees and among provenances compared with expression variations in the transcriptome. In contrast, we observed a substantial constitutive differentiation in gene expression activity related to photosynthesis and secondary metabolism between both provenances. Therefore we assume that local adaptation in *Pseudotsuga menziesii* is unlikely to be driven by divergent transcriptional short-term responses to environment among populations, suggesting that local adaptation is not reflected in short-term responses and instead determines long term physiological and metabolic processes.

## Methods

### Experimental design and plant material

Two Douglas-fir provenances originating from the western pacific coast of North America, Cameron Lake (LA) and westwards from the Rocky Mountains, Salmon Arm (AR) were investigated. While Cameron Lake represents a coastal Douglas-fir originating from Vancouver Island, Salmon Arm originates from an inland hybridization zone of coastal and interior Douglas-fir. The origins are in relative proximity (~ 1000 km) and vary by one degree in latitude. The origins differ in elevation (AR: 650 m, LA: 210 m), mean annual temperature (AR: 7.8 °C, LA: 10 °C) and most importantly in mean annual precipitation (AR: 500 mm, LA: 1475 mm). Recent studies using SNP [77] and microsatellite markers [78] revealed a clear genetic differentiation of these two provenances. For this study we used 50-year-old Dougls-fir trees from two common garden experiments near Schluchsee (S; 47°84′ N, 8°11′ E) and Wiesloch (W; 49°30′ N, 5°53′ E) in south-western Germany. The trees were planted during the International Douglas-fir provenance trial of 1958 [79]. The two sites differ in annual precipitation (S: 1345 mm, W: 660 mm) as well as in elevation (S: 1050 m, W: 105 m above sea level) and annual mean temperature (S: 6.1 °C, W: 9.9 °C). A detailed description of the provenances and the two field sites can be found in [78, 80]. At each field site needle samples were taken repeatedly from 8 trees per provenance and on four different dates during the 2010 growing season (in Schluchsee on May 27, June 30, July 28, September 15; in Wiesloch on May 12, June 16, July 14, September 8; these eight sampling dates reflect the levels of the factor *DATE* that was used for modeling of gene expression, see below). This resulted initially in a total of 128 samples. Subsequent quality testing in the lab revealed variation in RNA quality. Excluding samples with RNA that was not suitable for RNA sequencing resulted in a final number of 75 needle samples that were used to generate libraries for RNA sequencing. A detailed overview listing all trees and samples included in this study is provided in Additional file *5*: Table S3. Needle samples were collected from the upper sun exposed southern canopy (~ 3 m below the top, at a height of about 25–30 m). Previous year’s needles (2009) were collected around noon (12:30–15:00) and immediately frozen in liquid nitrogen upon collection.

### Library preparation and Illumina mRNA sequencing

After homogenization in liquid nitrogen using mortar and pestle, total RNA was extracted using a CTAB based extraction method, modified after [81]. After precipitation and resuspending the RNA, a DNase I digestion was performed, using Qiagens RNase-Free DNase Set (Cat. no. 79254). Afterwards, the DNase was removed using silica columns (Qiagen RNEasy MinElute, Cat. no. 74204). The integrity of the total RNA was checked on a 2100 Bio-analyzer (Agilent, CA, USA) using the RNA 6000 nano assay and the Plant total RNA protocol. The purity of total RNA was checked on a Nanodrop ND-1000 (Thermo Scientific, Bremen, Germany). From each sample, one deep mRNA sequencing library was prepared, using the TruSeq RNA Sample Preparation Kit v2 starting from 4 μg total RNA (Illumina, CA, USA). The libraries were prepared and sequenced on two Illumina 100 bp paired end (PE) flow cells on the Illumina HISEQ 2000 at the Genome Quebec Innovation Centre in Montreal, Canada. Based on earlier investigations [82], we aimed at an effective sequencing depth of 20 million aligned reads per sample.

### Alignment to joint Douglas-fir PUT set

We merged the recently published putatively unique transcript (PUT) sets for Douglas-fir [46, 47] to create a non-redundant set of PUTs using CD-HIT-EST (Version 4.6) [83], a tool for fast clustering of nucleotide and protein sequences. PUTs that were entirely covered by longer PUTs and have 99 % sequence identity with the longer PUT were removed. Finally all PUTs smaller than 200 bp were discarded. The 100 bp paired-end (PE) reads from the Illumina sequencing were aligned to the joint Douglas-fir PUT-set with Bowtie2 (Version 2.1.0) [84] using the global alignment mode and otherwise default settings. The alignment was parsed and analyzed with a custom Python script which builds on the HTSeq python library [85]. All hits below a threshold sequence identity of 95 % and alignment length of 80 were discarded. When multiple best hits existed, i.e. hits with the same sequence identity and the same alignment length, one of these hits was randomly selected by Bowtie2. The PUT set contains potential splice variants. Splice variants that are anticipated to emerge from a common genomic locus are grouped in one isogroup and sequence stretches may appear multiple times in the PUT set. Thus, uniqueness of a hit was not considered. Subsequently, reads that aligned to a PUT were counted and converted to counts per PUT using a custom Python script. Only PUTs with more than 1 read per million aligned reads (RPM) in at least four libraries were retained.

### Functional annotation of PUTs

To functionally annotate the detected PUTs, we did a BLASTX search in the *Arabidopsis thaliana* peptide data base (TAIR 10) and the NCBI Plant RefSeq peptide data base (date of download: May-8-2013) (BLASTX, NCBI BLAST+ suite, Version 2.2.24+, E < 1e-3, sequence identity >40 % ). For Gene Ontology (GO) [86] annotation, the results of the BLASTX search against the NCBI plant RefSeq proteins (RefSeq) were fed into the Blast2GO pipeline [87]. Functional annotation is also described in [82]. The detected PUTs were also aligned to a high quality mostly full length *Picea glauca* EST set [48], to a set of transcripts of *Pinus taeda* which has been used to annotate the *Pinus taeda* genome [88] and to *Vitis vinifera* and *Oryza sativa* peptides stored in the PLAZA data base (Version 2.5) [89] using RAPSEARCH [90]. RAPSEARCH is similar to BLAST but uses a reduced amino acid alphabet to increase processing speed and the identification of seeds in the query sequences. It is thus 20 to 90 times faster than BLAST at the drawback of a slightly reduced sensitivity [90]. The reduced sensitivity results in missing alignments with high E-values. We retained alignments identified by RAPSEARCH with E-value < 1e-5. The expressed PUTs were assigned to gene families stored in the PLAZA data base using the TRAPID functional annotation pipeline [91].

### Data transformation and exploratory analysis

#### Normalization for sequencing library size and variance stabilizing transformation

Differences in library size between the deep sequencing libraries were corrected using the method implemented in the Bioconductor [92] package DESeq [93] which is one of the most robust methods to correct for library size [94]. Log transformation of the number of aligned reads per PUT (Fig. 1c) resulted in normally distributed data for more than 95 % of PUTs (Kolmogorov-Smirnov test, *P* > 0.05).

#### Estimation of variance components attributed to individual tree, provenance and environment

To estimate the amount of variance in PUT abundance that is attributed to each individual tree, provenance or environment, we estimated the respective variance components using a linear random effects model. We fit a model that included the individual tree (*TREE*), the common garden (*SITE*), the provenance (*PROVENANCE*) and the sampling time-point (*DATE*) to the transformed count data of each detected PUT using the R [95] package lme4 [96] and restricted maximum likelihood (REML). Variance components for each random effect were extracted and divided against the sum of all variance components and the residual variance.

### Linear modelling of PUT abundance

#### Description of environmental parameters and genetic factors as regressors for the linear regression models

Total available soil water (*TAW*), air temperature (*TEMPERATURE*) and day length (*DAYLENGTH*) were used as environmental parameters in our analysis. *TAW* was obtained from the forest water model WBS3 [97] which uses mean daily temperature and daily precipitation as the meteorological input parameters, combined with latitude, soil type, plant cover, slope and slope aspect. Mean daily temperature and mean daily precipitation were measured at two weather stations, one operated by “Deutscher Wetterdienst” (DWD) close to the field site Wiesloch and one privately operated close to the field site Schluchsee. *TEMPERATURE* in our analysis represents the average air temperature of the sampling day recorded between 10:00 am and 2:00 pm centered to the four week running average of air temperature. Centering was performed to detrend the temperature which is correlated with day length and to identify time-points of above-average temperature. Day length (*DAYLENGTH*) on the day of sampling was centered to the length of the longest day of the year (solstice on June 21). To account for the season and the direction of an increasing versus a decreasing day length before and after solstice, day length before June 21 was assigned a positive value and after June 21 a negative value. The field site and the provenance were encoded as dummy variables (*SITE* and *PROVENANCE*, respectively).

#### Detection of differential expression

Differential PUT expression was investigated using linear mixed models. Models were fit to the log-transformed count data using the function lmer in lme4 [96]. We compared nested models using an F-test with Kenward-Roger approximation implemented in the R-package pbkrtest. *P*-values from the F-test were adjusted for multiple testing using the Benjamini-Hochberg procedure. In addition to the F-test the dAIC and dR^2^ were calculated as the difference in Akaike information criterion (AIC) and coefficient of determination (R^2^) of the model containing the regressor compared to the model without it (Fig. 1e). The R^2^ for the fixed effects part of the model was calculated according to [98].

$$ {R}_{marg}^2=\frac{\sigma_{fix}^2}{\sigma_{fix}^2+{\sigma}_{rand}^2+{\sigma}_{err}^2} $$

(σ^2^_fix_ = variance attributed to fixed effects, σ^2^_rand_ = variance attributed to random effects, σ^2^_err_ = residual variance).

To test for differential expression between provenances and sampling time-points (*DATE*) including interaction of both we set up and compared four models (Fig. 1e). Each of the models contained *TREE* as random intercept. After testing for the interaction effect it was removed from the model and the effects of *DATE* and *PROVENANCE* were tested. Differential expression in response to the environmental parameters represented by *TEMPERATURE*, *DAYLENGTH* and *TAW* was investigated by removing a single environmental parameter from the model *TAW* + *TEMPERATURE* + *DAYLENGTH* + *PROVENANCE* and comparing both models. PUTs are only considered to respond to an environmental parameter if the corresponding regressor increases the R^2^ by more than 0.2, in addition to an FDR smaller than 0.01. Multicollinearity (variance inflation factor >10) did not allow for introducing water availability (*TAW*) and common garden (*SITE*) simultaneously into the model (Additional file 1: Figure S1), thus we did not include *SITE*. We did not consider interactions among the environmental parameters to avoid over fitting due to the limited number of sampling points (*n* = 8).

### Identification of Gene Ontology (GO) category overrepresentation

GO categories were defined as significant, if they are overrepresented in PUTs that respond to a regressor (*p* < 0.01, Fisher exact test). Furthermore, we required significant GO categories to contain more than ten PUTs that responded to a regressor. Overrepresentation analyses were conducted using the Bioconductor package topGO [99].

The PUTs investigated in our analysis are assembled EST sequences and therefore contain various isoforms of a gene product as well as incompletely assembled sequences, although overrepresentation analyses by GO categories assume a single gene locus. Therefore, we reduced the risk of artificially inflated abundance of annotations by randomly selecting one PUT from all PUTs that shared a best hit in the NCBI plant RefSeq data base. We repeated the overrepresentation analysis 100 times and averaged the results because different PUTs with the same best BLAST hit might not equally respond to a regressor.

For visualization of the results of the GO analysis, GO categories were grouped according to the amount of overlapping PUTs among two GO categories. This overlap was identified based on the Kappa statistic (Cohen’s Kappa).

$$ \kappa =\frac{O_{mn}-{A}_{mn}}{1-{A}_{mn}} $$

Clustering of genes based on the Kappa statistic has been described by [100]. In brief the number of shared PUTs among two GO categories is expressed by the Kappa statistic calculated from presence absence matrices (0 / 1) where rows correspond to GO categories and columns correspond to PUTs. Omn corresponds to the co-occurrence of PUTs in GO categories m and n while Amn represents the co-occurrence of PUTs in GO categories m and n expected by chance. In contrast to [100] who employed a heuristic fuzzy partition algorithm, we identified clusters of GO categories which shared PUTs by hierarchical clustering on distance matrices created from the Kappa scores (method “euclidean”). As basal GO terms do generally carry a low information content [101], we selected only GO categories with at least five parental terms.

## Additional files

Additional file 1: Figure S1. Relationship among environmental regressors. Color indicates the absolute value of Pearson’s correlation coefficient calculated for each regressor pair. TAW = Total available soil water, SITE = common garden, TEMPERATURE. DAYLENGTH = interaction of TEMPERATURE and DAYLENGTH, TAW. DAYLENGTH = interaction of TAW and DAYLENGTH, TAW. TEMPERATURE = interaction among TAW and TEMPERATURE. (PDF 333 kb) Additional file 2: Table S1. Results of the overrepresentation analysis (*P*-value Fisher’s exact test <0.01). *P*-value Fisher’s exact test = *P*-value from the overrepresentation analysis, Expected = expected frequency of the GO category, given the abundance of the GO category in the set of all detected PUTs, Significant = observed frequency of GO category. Results are ranked based on the *P*-value of the overrepresentation test (Fisher’s exact test). (XLS 105 kb) Additional file 3: Table S2. Functional annotation of differentially expressed PUTs within overrepresented GO categories. *P*-values from the test of differential expression were averaged over all PUTs (PUTs) with the same best hit in the RefSeq data base. Annotations are ranked (Rank) according to the absolute log10 transformed *P*-value (p_Value_log) from the test of differential expression. Accession = RefSeq ID of the best hit in the RefSeq data base, GI = NCBI Entrez ID of the best hit in the RefSeq data base, Ara = best hit in the *Arabidopsis thaliana* peptide data base (TAIR10), Annot = annotation inferred by the Blast2GO pipeline, Ara ID = *Arabidopsis thaliana* gene id, GO Reference = GO annotation shown in Fig. 6, PUTs = significant PUTs, separated by “--”, BLAST TAIR10 = e-value of the best hit in the TAIR10 peptide database inferred by a BLASTX search for the corresponding PUT, separated by “--”, BLAST RefSeq = e-value of the best hit in the RefSeq database inferred by a BLASTX search for the corresponding PUT, separated by “--”. (XLSX 738 kb) Additional file 4: Figure S2. Expression of the 21 PUTs with significant GxE interaction effect (FDR <0.01). Expression is shown for each time-point on each common garden. Number of aligned reads were normalized for sequencing depth and log10 transformed. Whiskers of boxplots extend to 1.5 times the interquartile range, dots represent outliers. Cameron Lake = cyan color, Salmon Arm = magenta. (PDF 50 kb) Additional file 5: Table S3. Detailed information about the sample characteristics. (XLS 36 kb)

## Abbreviations

AIC: Akaike information criterion
AR: Salmon Arm
dAIC: delta AIC
dR^2^: delta R2
DWD: Deutscher Wetterdienst
E: Environment
FDR: False discovery rate
G: Genotype
GO: Gene ontology
GxE: Gene by environment
k: 1000
LA: Cameron Lake
LMM: Linear mixed model
Mread: Mega reads
PE: Paired end
PUT: Putative unique transcript
REML: Restricted maximum likelihood
RPM: Reads per million aligned reads
S: Schluchsee
*TAW*: Total available soil water
TMM: Trimmed mean of M-values
W: Wiesloch
WUE: Water use efficiency
