# Erratum to: Transcriptome responses to temperature, water availability and photoperiod are conserved among mature trees of two divergent Douglas-fir provenances from a coastal and an interior habitat

**DOI:** 10.1186/s12864-016-3144-x

**Published:** 2016-10-06

**Authors:** Moritz Hess, Henning Wildhagen, Laura Verena Junker, Ingo Ensminger

**Affiliations:** 1 Forest Research Institute of Baden-Württemberg (FVA), Wonnhaldestrasse 4, D-79100 Freiburg i. Brsg., Germany; 2 Institute for Biology III, Faculty of Biology, Albert Ludwigs University Freiburg, Schänzlestrasse 1, D-79104 Freiburg i. Brsg., Germany; 3 Department of Biology, Graduate Programs in Cell & Systems Biology and Ecology & Evolutionary Biology, University of Toronto, 3359 Mississauga Road, Mississauga, ON L5L 1C6 Canada; 4 Present Address: Department of Forest Botany and Tree Physiology, Büsgen-Institute, Georg-August-University Göttingen, Büsgenweg 2, D-37077 Göttingen, Germany; 5 Present Address: Institute of Medical Biometry, Epidemiology and Informatics (IMBEI), University Medical Center Mainz, Obere Zahlbacher Strasse 69, 55131 Mainz, Germany

## Erratum

In the original publication of this article [1], a formatting field was mistakenly deleted, changing the entire composition of the first formula listed in the article.

The following formula is incorrect:

$$ \frac{R_{m \arg}^2={\sigma_{fix}}^2}{{\sigma_{fix}}^2+{\sigma}_{rand}^2+{\sigma}_{err}^2} $$

This should have been displayed as:

$$ {R}_{marg}^2=\frac{\sigma_{fix}^2}{\sigma_{fix}^2+{\sigma}_{rand}^2+{\sigma}_{err}^2} $$
