# Supplementary material for: Transcriptome responses to temperature, water availability and photoperiod are conserved among mature trees of two divergent Douglas-fir provenances from a coastal and an interior habitat
Source: BMC Genomics. 2016 Aug 26;17(1):682. doi: 10.1186/s12864-016-3022-6 (PMC5002200; doi:10.1186/s12864-016-3022-6)
Supplement: Additional file 1: Figure S1. — Relationship among environmental regressors. Color indicates the absolute value of Pearson’s correlation coefficient calculated for each regressor pair. TAW = Total available soil water, SITE = common garden, TEMPERATURE. DAYLENGTH = interaction of TEMPERATURE and DAYLENGTH, TAW. DAYLENGTH = interaction of TAW and DAYLENGTH, TAW. TEMPERATURE = interaction among TAW and TEMPERATURE. (PDF 333 kb) [file 12864_2016_3022_MOESM1_ESM.pdf]

Figure S1

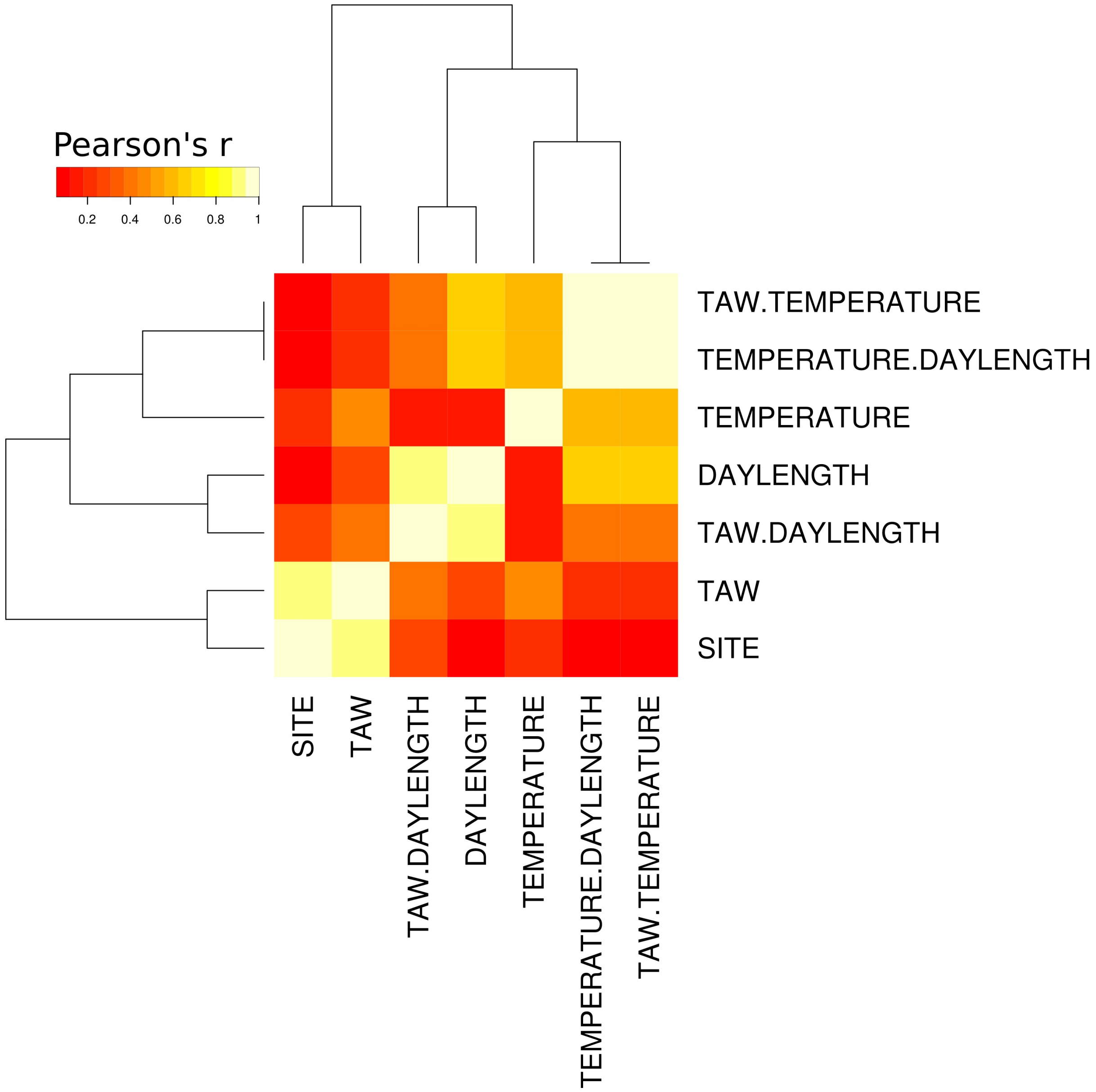

Figure S1: Relationship among environmental regressors

Color indicates the absolute value of Pearson's regression coefficient calculated for each regressor pair. TAW = Total available soil water, SITE = common garden, TEMPERATURE.DAYLENGTH = interaction of TEMPERATURE and DAYLENGTH, TAW.DAYLENGTH = interaction of TAW and DAYLENGTH, TAW.TEMPERATURE = interaction among TAW and TEMPERATURE.
