# Supplementary material for: Transcriptome responses to temperature, water availability and photoperiod are conserved among mature trees of two divergent Douglas-fir provenances from a coastal and an interior habitat
Source: BMC Genomics. 2016 Aug 26;17(1):682. doi: 10.1186/s12864-016-3022-6 (PMC5002200; doi:10.1186/s12864-016-3022-6)

isotig22235|−−|−−

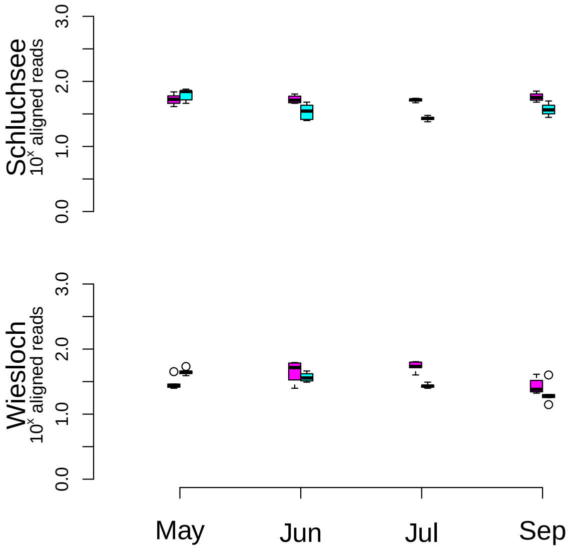

isotig69238|−−|−−

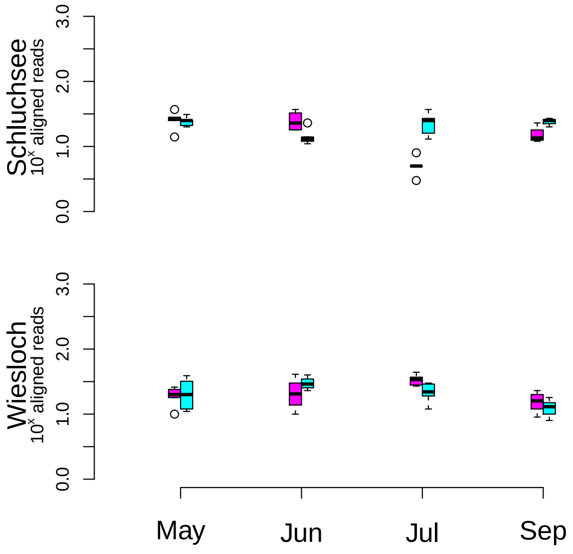

GAEK01022239.1|−−|−−

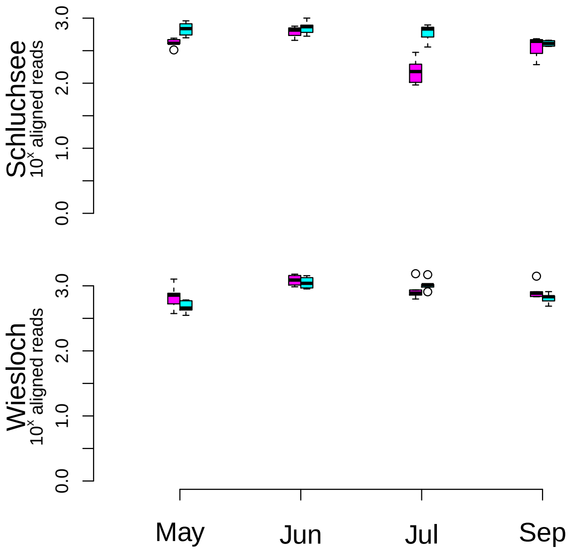

GAEK01036740.1|zinc finger ccch domain−containing protein 13−like|−−

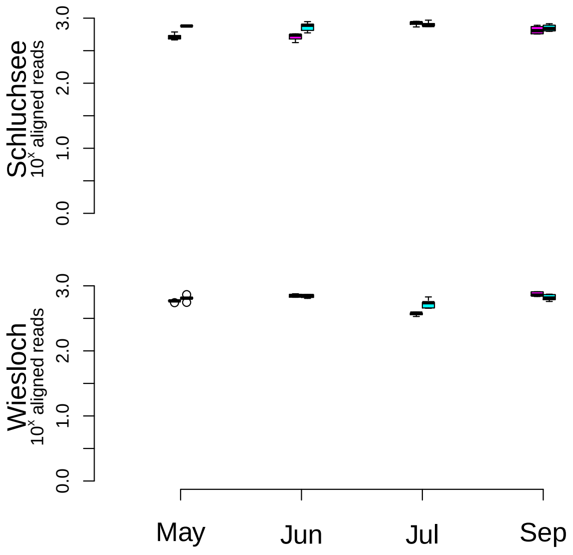

isotig87523|−−|−−

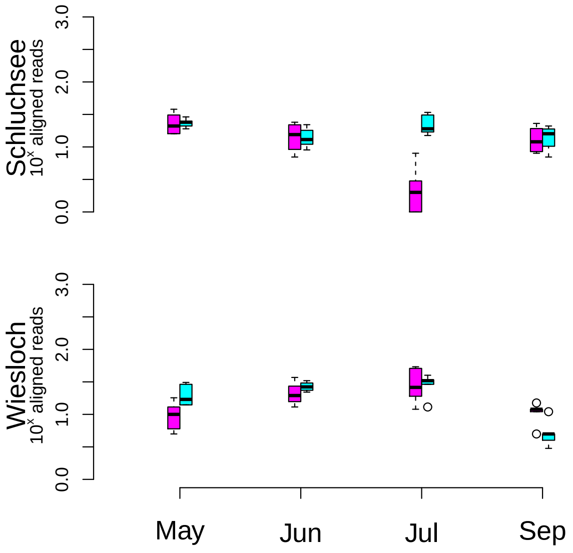

isotig04818|−−|−−

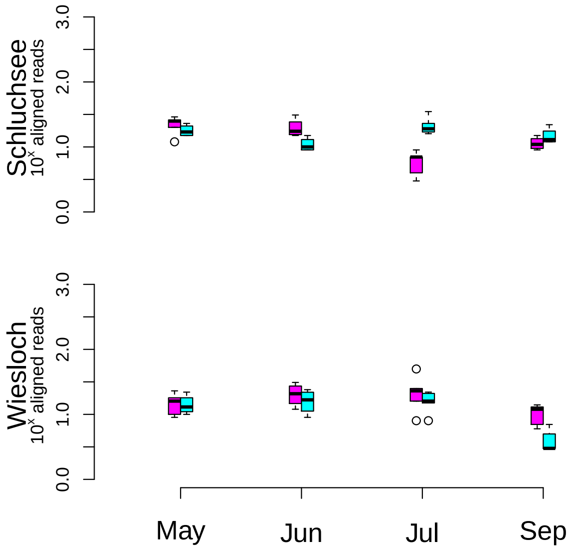

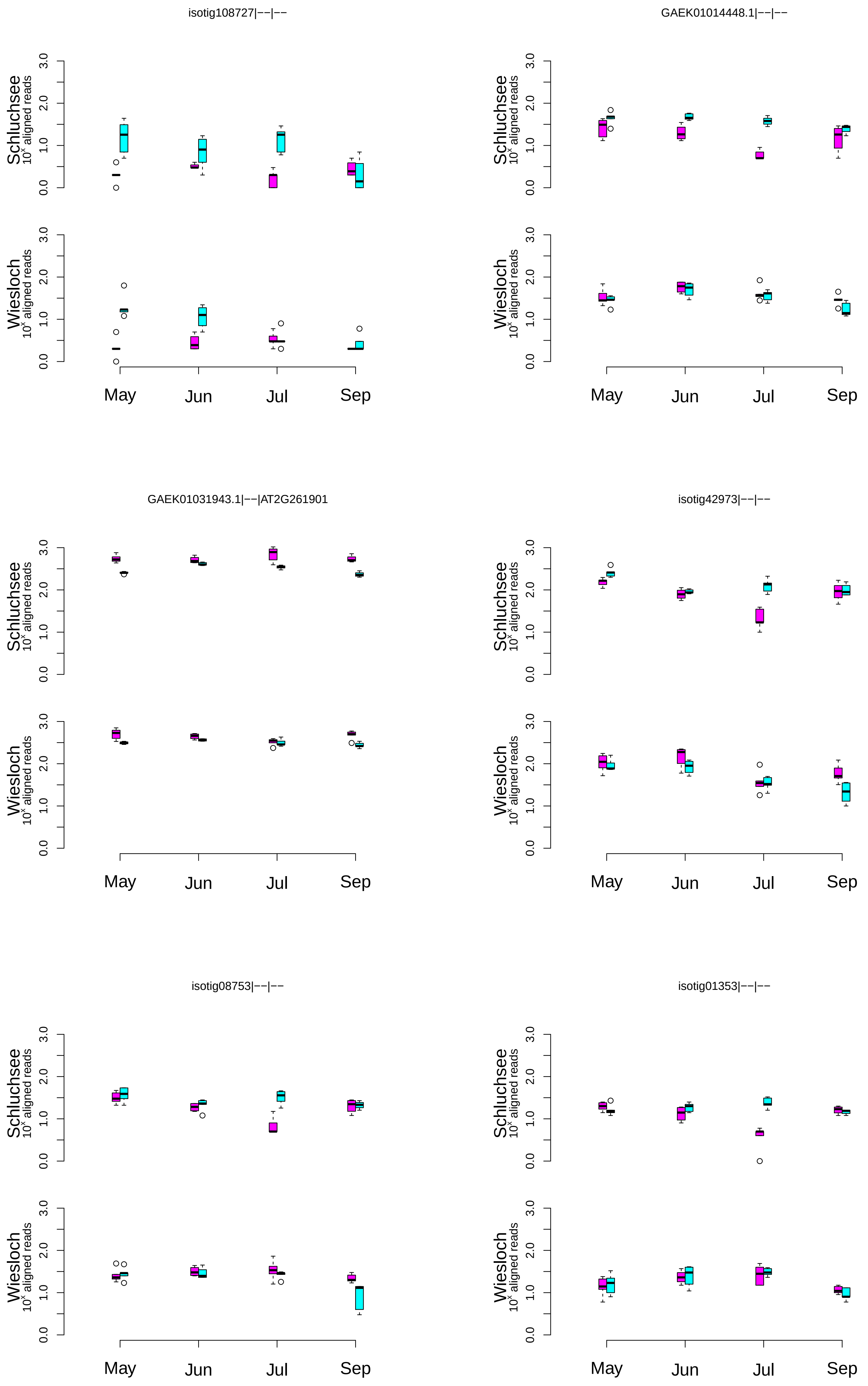
isotig108727|−−|−−
GAEK01014448.1|−−|−−
GAEK01031943.1|−−|AT2G261901
isotig42973|−−|−−
isotig08753|−−|−−
isotig01353|−−|−−
Schluchsee
Wiesloch
10^x aligned reads
0.0
1.0
2.0
3.0
May
Jun
Jul
Sep

isotig71268|−−|−−

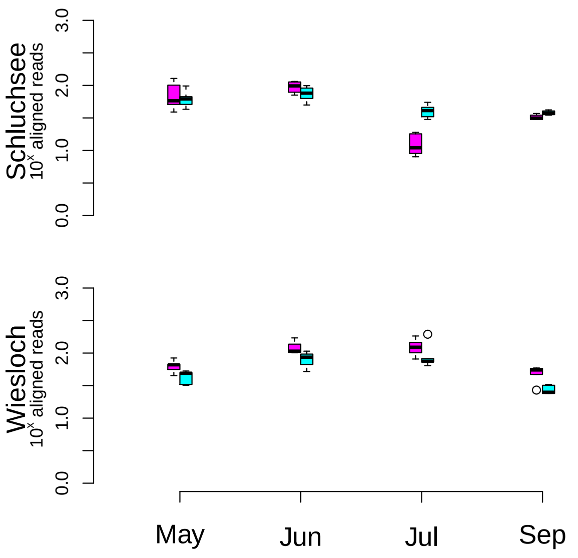

isotig57365|−−|−−

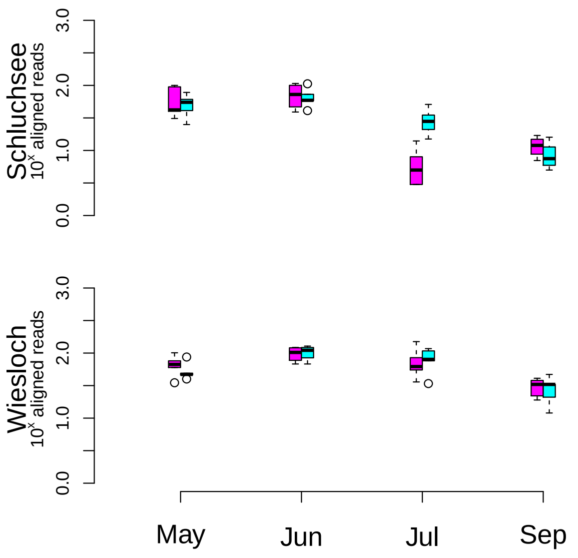

diwc_J01DAN2D|−−|−−

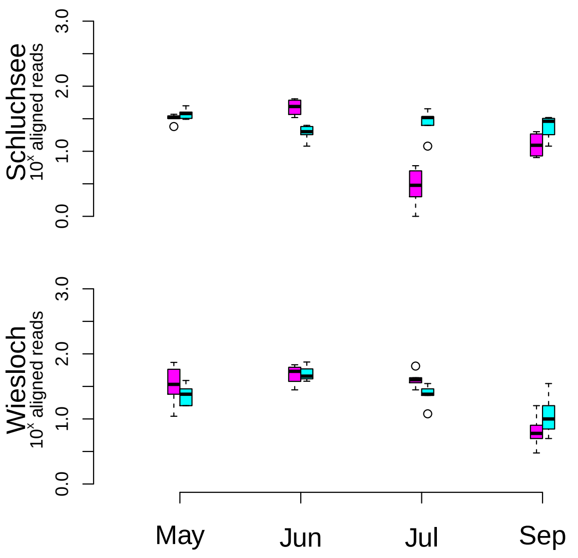

isotig19825|−−|−−

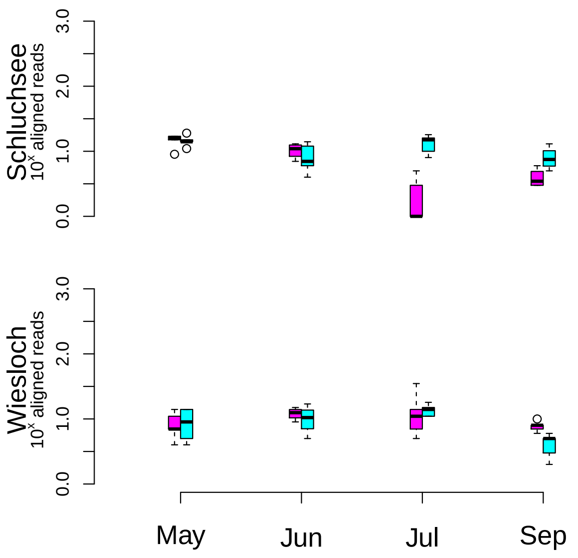

isotig12634|uncharacterized protein loc100780312|−−

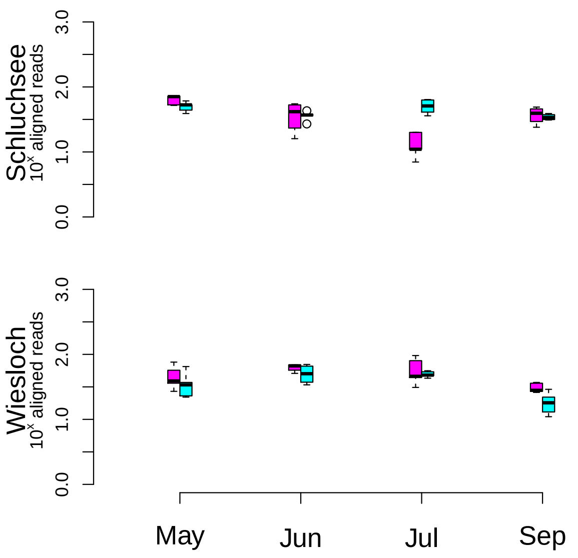

isotig15364|−−|−−

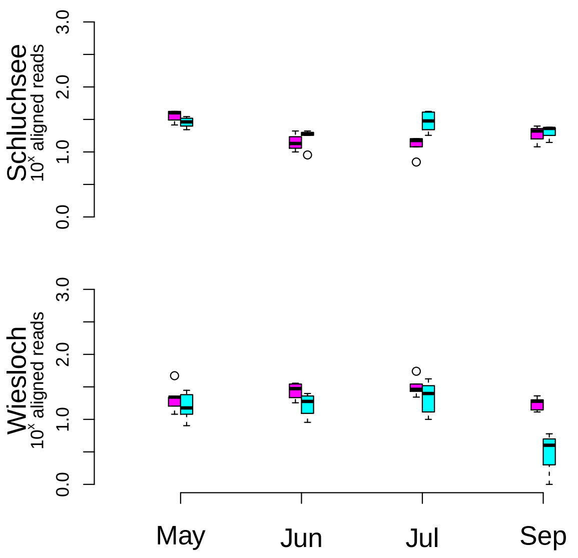

GAEK01020103.1|uncharacterized protein loc101245822|AT4G349401

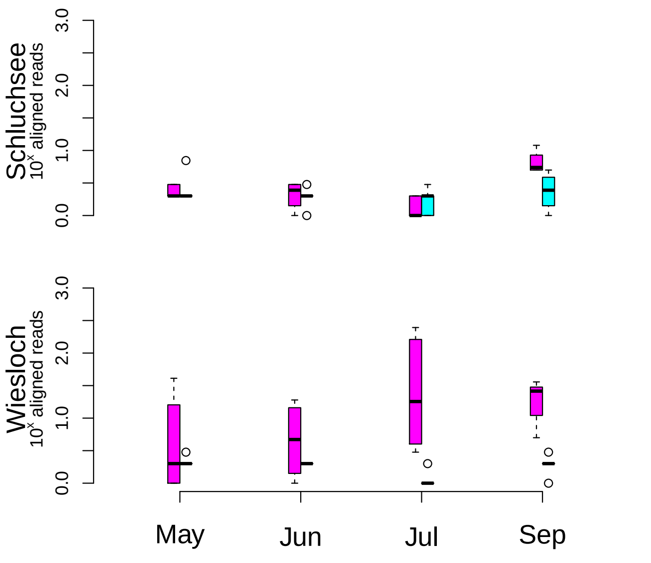

GAEK01021507.1|--|--

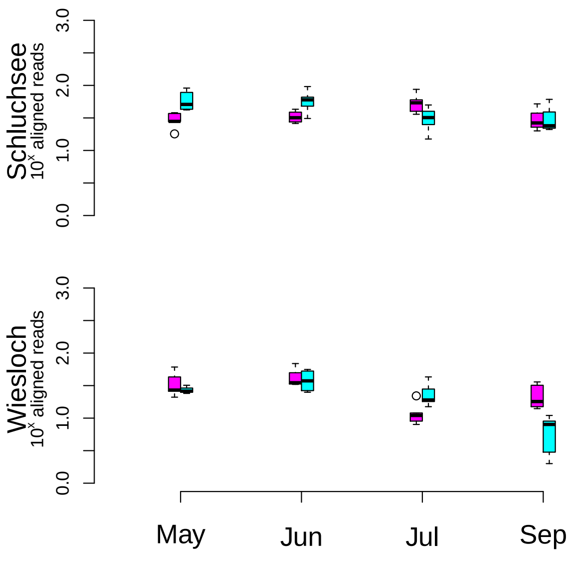

isotig51556|cytochrome c oxidase subunit 1|ATMG013601

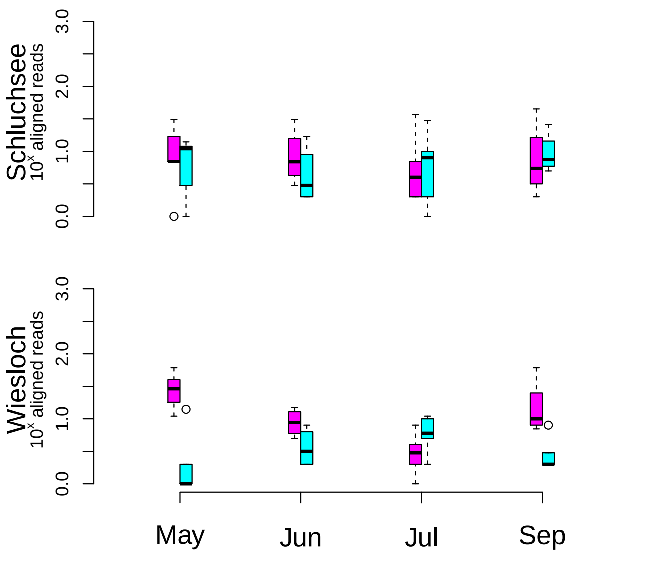

Supplement: Additional file 4: Figure S2. — Expression of the 21 PUTs with significant GxE interaction effect (FDR <0.01). Expression is shown for each time-point on each common garden. Number of aligned reads were normalized for sequencing depth and log10 transformed. Whiskers of boxplots extend to 1.5 times the interquartile range, dots represent outliers. Cameron Lake = cyan color, Salmon Arm = magenta. (PDF 50 kb) [file 12864_2016_3022_MOESM4_ESM.pdf]
